# The Pivotal Role of Semantic Memory in Remembering the Past and Imagining the Future

**DOI:** 10.3389/fnbeh.2013.00027

**Published:** 2013-04-03

**Authors:** Muireann Irish, Olivier Piguet

**Affiliations:** ^1^School of Psychology, University of New South WalesSydney, NSW, Australia; ^2^Neuroscience Research AustraliaRandwick, NSW, Australia; ^3^Australian Research Council Centre of Excellence in Cognition and its DisordersSydney, NSW, Australia; ^4^School of Medical Sciences, University of New South WalesSydney, NSW, Australia

**Keywords:** semantic dementia, autobiographical memory, future thinking, Alzheimer’s disease, episodic memory, anterior temporal lobes, semantic memory

## Abstract

Episodic memory refers to a complex and multifaceted process which enables the retrieval of richly detailed evocative memories from the past. In contrast, semantic memory is conceptualized as the retrieval of general conceptual knowledge divested of a specific spatiotemporal context. The neural substrates of the episodic and semantic memory systems have been dissociated in healthy individuals during functional imaging studies, and in clinical cohorts, leading to the prevailing view that episodic and semantic memory represent functionally distinct systems subtended by discrete neurobiological substrates. Importantly, however, converging evidence focusing on widespread neural networks now points to significant overlap between those regions essential for retrieval of autobiographical memories, episodic learning, and semantic processing. Here we review recent advances in episodic memory research focusing on neurodegenerative populations which has proved revelatory for our understanding of the complex interplay between episodic and semantic memory. Whereas episodic memory research has traditionally focused on retrieval of autobiographical events from the past, we also include evidence from the recent paradigm shift in which episodic memory is viewed as an adaptive and constructive process which facilitates the imagining of possible events in the future. We examine the available evidence which converges to highlight the pivotal role of semantic memory in providing schemas and meaning whether one is engaged in autobiographical retrieval for the past, or indeed, is endeavoring to construct a plausible scenario of an event in the future. It therefore seems plausible to contend that semantic processing may underlie most, if not all, forms of episodic memory, irrespective of temporal condition.

## Introduction

**Figure 1:**
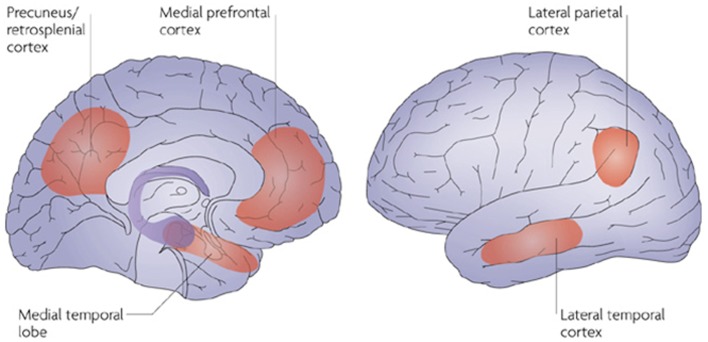
**Component structures of the core memory network consistently activated during autobiographical retrieval of the past, and constructive simulation of the future**. Notably, the lateral temporal cortex is implicated across past and future contexts, highlighting the central role for semantic memory in past and future oriented thought. Reprinted by permission from Macmillan Publishers Ltd., Schacter et al. (2007). Copyright (2007).

One of the most fascinating aspects of human cognition is our ability to withdraw from the current moment and to mentally transport ourselves to another time, place, or perspective. Collectively, the abilities to remember the past via episodic autobiographical memory (ABM), or to imagine possible future events, represent important expressions of the human memory system (Tulving, 2002), potentially conferring a significant adaptive advantage in planning for the future (Suddendorf and Corballis, 2007; Klein, 2013). In recent years, episodic memory has been reconceptualized as not only the capacity for retrieval from our personal past, but also encompassing the ability to imagine and envisage possible future scenarios, leading to a constructivist view on how humans might achieve such sophisticated acts of cognition (Hassabis and Maguire, 2007; Schacter and Addis, 2007a). Neuroimaging studies have uncovered a widespread “core” network which subtends the successful retrieval of autobiographical memories from our past (Maguire, 2001; Svoboda et al., 2006; Cabeza and St. Jacques, 2007; Schacter et al., 2007). Importantly, this core network includes frontal and medial temporal regions, notably the hippocampus, lateral temporal, sensory association cortices, and more posterior parietal regions (Spreng et al., 2009), reflecting the multifaceted nature of this type of memory (see Figure 1). While considerable variation exists in the classification of episodic memory, in this article, we refer to the contents of episodic memory as “remembered experiences,” or ABM (see Tulving and Szpunar, 2009). As will become evident, however, the contents of episodic memory invariably involve semantic representations.

In contrast to the largely evocative, spatiotemporally specific, and often emotionally charged, instances of episodic memories from the past sits the repository of all acquired atemporal knowledge of the world, semantic memory. Traditionally, the episodic/semantic distinction has served as a useful heuristic within the neuropsychological literature (Greenberg and Verfaellie, 2010), underscoring the importance of the medial temporal lobes, specifically the hippocampus, in the encoding and retrieval of episodic memories in contrast with the centrality of the anterior temporal lobes for the retrieval of semantic information. Similar to episodic memory, semantic memory is viewed as essential for many aspects of cognition, including language, reasoning, planning, problem-solving, and social interaction (Binder et al., 2009). In support of this position, neuroimaging studies have demonstrated significant activation in semantic processing regions in healthy individuals across a wide variety of cognitive abilities including distinguishing real from fictitious scenarios (Abraham et al., 2008a), positive counterfactual thinking (Van Hoeck et al., in press), as well as engaging in processes relevant for creativity (Vartanian, 2012). In contrast to the spatiotemporal specificity of episodic memory, semantic memory is derived from the abstraction of content from experiences, which is represented using modality-specific simulations whereby information relevant to a specific mode of experience is processed within the corresponding sensory, motor, or affective system (Binder and Desai, 2011). These representations are processed, therefore, in high level supramodal convergence zones in the brain including the inferior parietal cortex, the middle and inferior temporal gyri, and anterior portions of the fusiform gyrus (Patterson et al., 2007; Binder et al., 2009; Binder and Desai, 2011). Importantly, the semantic system recently identified in a meta-analysis of 120 functional neuroimaging studies exhibits striking overlap to the large-scale core ABM network (Maguire, 2001; Svoboda et al., 2006; Binder et al., 2009) prompting the observation that autobiographical memories necessarily contain a high level of semantic concepts (Binder et al., 2009), and that, by its nature, semantic representations are essential for a host of complex cognitive functions including remembering the past and imaging the future (Binder and Desai, 2011). These recent proposals resonate with mounting evidence from the neuroimaging literature demonstrating considerable overlap between episodic and semantic memory systems and the unclear boundaries that exist between these forms of memory (Burianova et al., 2010).

With the advent of sophisticated neuroimaging techniques, we have witnessed a shift in perspective from studying specific brain structures in isolation to the consideration of carefully orchestrated neural networks. Converging evidence now points to the role of large-scale neural networks in subtending complex cognitive processes, and the intriguing possibility that semantic processing may play a central role in all aspects of internal mentation.

## The Study of Neurodegenerative Disorders

Neurodegenerative disorders offer a compelling view of the cognitive architecture of the brain when specific neural systems break down in a coordinated fashion (Irish et al., 2012c). In this review, we will focus on Alzheimer’s disease (AD) and semantic dementia (SD) as lesion models for episodic and semantic memory processes, respectively, to demonstrate how these dementia syndromes illuminate our understanding of the complexity of the episodic memory system, and crucially, how episodic and semantic memory invariably interact during complex forms of past and future oriented thought.

Alzheimer’s disease has long been heralded as a suitable lesion model for episodic memory processes, in light of the characteristic medial temporal lobe neural degeneration evident from a very early stage in the pathological process (Butters et al., 1987; Braak and Braak, 1991). Recent studies point to the preferential accumulation of amyloid deposits in specific nodes of the core ABM network in AD, most notably the posterior cingulate cortex and the anteromedial prefrontal cortex (Buckner et al., 2008). Clinically, AD patients typically present with an amnestic profile in which anterograde episodic memory difficulties concerning the encoding and retrieval of recent events are prominent (de Toledo-Morrell et al., 2000; McKhann et al., 2011). This disruption to episodic memory emerges as a consequence of the neuropathological process (neurofibrillary tangles and amyloid deposition), which affects the entorhinal cortex and hippocampus of the medial temporal lobes, and spreads to the neocortex (Ewers et al., 2011; Sperling et al., 2011). Importantly, such episodic memory deficits occur in the context of a relative sparing of semantic processing in the early stages of the disease (see Table 1).

**Table 1:** **Clinical characteristics of semantic dementia and Alzheimer’s disease**.

	Semantic dementia (SD)	Alzheimer’s disease (AD)
**Predominant brain atrophy**	Lateral temporal Left > Right	Medial temporal Bilateral
**Cognition**^a^		
Executive function	Intact	+ – ++
Orientation	Intact	++
Recent episodic memory	Intact^b^	+++
Remote episodic memory	+++	+
Semantic knowledge	+++	±
Word comprehension	+++	±

*^a^Functions are relatively intact in the early stages of the disease; ^b^contingent on the nature of test materials used; +, mild deficits; ++, moderate deficits; +++, severe deficits; ±, variable deficits*.

The neurodegenerative disorder of SD represents the other side of the coin, in that the hallmark clinical feature of this disease concerns the progressive and amodal loss of semantic or general conceptual knowledge of the world (Hodges and Patterson, 2007). This loss of world knowledge occurs irrespective of modality and is theoretically attributable to the deterioration of a central amodal semantic hub (Rogers et al., 2004; Patterson et al., 2007; but, see Mesulam et al., 2013). On a neural level, SD is characterized by the progressive degeneration of the anterior temporal lobes (Hodges and Patterson, 2007), most severe on the ventral surface and including the perirhinal cortex, anterior fusiform gyrus (Whitwell et al., 2005; Mion et al., 2010), and typically lateralized to the left more than the right hemisphere. Importantly, volumetric MRI studies have confirmed that the degree of hippocampal atrophy in SD is equivalent, or greater, to that seen in disease-matched cases of AD, albeit in the context of much more severe temporal lobe atrophy (Chan et al., 2001; Galton et al., 2001). Of paramount importance in the current context, however, is the observation that despite profound semantic deficits, SD patients nevertheless display otherwise relatively preserved cognitive functions, including retrieval of recent episodic information, particularly when non-verbal tasks are employed (see Table 1; Bozeat et al., 2000; Crutch and Warrington, 2002; Hodges and Patterson, 2007).

In summary, the neurodegenerative disorders of AD and SD offer a unique opportunity to disentangle the interaction between the episodic and semantic memory systems. A theoretically important distinction is evident: while in AD we see the loss of episodic memory in the context of medial temporal lobe degeneration and relative preservation of semantic knowledge, in SD, the amodal deterioration of semantic memory occurs in the context of relatively preserved recent episodic memory. A range of interdependencies between episodic and semantic memory have recently been expounded (Greenberg and Verfaellie, 2010). Here, however, we will constrain our focus to explore how these dementia syndromes inform our understanding of two putative expressions of the episodic memory system, namely autobiographical retrieval of the past, and simulation of the future.

## Remembering the Past – Autobiographical Memory

Perhaps the most important advances in understanding the interplay between episodic and semantic elements stem from the domain of ABM. The recollection of personal past memories from across our subjective timeline represents a powerful expression of the episodic memory system, requiring the retrieval of sensory-perceptual details, and emotional connotations, integrated within a specific spatiotemporal and personally relevant framework (Conway et al., 2004). Unsurprisingly, this complex endeavor is subtended by a distributed neural network involving the medial temporal lobes including the hippocampus and parahippocampal gyrus, the frontal poles, and more posterior regions including the posterior cingulate and parietal cortices, as well as the lateral temporal cortices (Maguire, 2001; Addis et al., 2004; Svoboda et al., 2006). It is noteworthy that across studies of ABM retrieval, activation of the lateral temporal cortices, regions known to be essential for semantic memory (Mion et al., 2010), is reliably observed (Spreng et al., 2009), suggesting a fundamental role for semantic processing underlying all forms of episodic past retrieval (see Figure 1).

## Preservation of Remote ABMs in Alzheimer’s Disease

The main structures implicated in ABM retrieval are those regions harboring significant atrophy in AD and SD. Importantly, our understanding of the neurocognitive mechanisms of ABM retrieval has been advanced from studying how the characteristic patterns of atrophy in AD and SD impact on the capacity for ABM retrieval. Little doubt exists regarding the prominent deficits in ABM typically seen in AD from early in the disease course. Irrespective of measure used, patients with AD demonstrate striking impairments, particularly on event or episodic subscales of these measures, in contrast with a relative preservation of personal semantics, at least in the early stages of the disease (Barnabe et al., 2012). A central debate in the ABM literature concerns the temporal profile of the episodic ABM deficit in AD, and specifically whether it conforms to Ribot’s law (Ribot, 1881), in which memories from more distant time periods appear relatively intact. A number of early studies of ABM have demonstrated a disproportionate impairment of recent compared to remote episodic memories in AD (Kopelman et al., 1989; Greene et al., 1995; Graham and Hodges, 1997; Eustache et al., 2004; Irish et al., 2006, 2011b; Leyhe et al., 2009), which in turn has been interpreted in favor of a time-limited role of the hippocampus in long-term retrieval (Squire and Alvarez, 1995). The preservation of remote memories in AD, however, is of interest if we consider that older memories are more likely to undergo a process of semanticization (Cermak, 1984), leading to overgeneral memories that are divested of rich episodic re-experiencing (Irish et al., 2008, 2011b). The relative sparing of the lateral temporal cortices, and a reasonably intact capacity for reminiscence in remote epochs of one’s life, in the early stages of AD accords with observations of reliance on gist memory in this syndrome (Gallo et al., 2006) and suggests that patients may overly depend on semantic representations of formerly episodic events. Similarly, patients with AD have been shown to lose access to sensory-perceptual details and appear particularly deficient in evoking specific self-referential visual imagery during ABM retrieval (Irish et al., 2011b). This loss of visual imagery may preclude the triggering of an emotional response (Kosslyn et al., 2001) and disrupts the overall re-experiencing of the retrieved event. A recent study has demonstrated that the capacity to generate complex visual imagery is compromised in AD, with the proposal that such deficits may impinge upon the envisaging of oneself across past and future contexts (Hussey et al., 2012). Thus, for AD patients, the retrieval of a past event occurs in the absence of vivid visual imagery, producing overgeneral and depersonalized or semanticized accounts of the formerly evocative event. These categories of events can be subsumed under Neisser’s concept of “repisodes” (Neisser, 1981), Barsalou’s “extended events” (Barsalou, 1988), or Conway’s view of “general events” within the ABM system (Conway, 2001; Greenberg and Verfaellie, 2010). While Irish et al. (2011b) reported a loss of self-referential visual imagery during retrieval of specific episodic autobiographical memories, the ability of AD patients to visualize repeated or abstracted experiences remains to be established. This represents an interesting, but underexplored area of research that has obvious relevance for the constructs under consideration.

Recent studies, including a large study from our group, have failed to demonstrate temporal gradients during ABM retrieval in AD (Piolino et al., 2003; Irish et al., 2011a). Such conflicting results may reflect differences in probing and scoring of ABMs across experimental protocols (Barnabe et al., 2012). Importantly, the separation of internal “episodic,” from external, or non-episodic, details using the Autobiographical Interview (AI; Levine et al., 2002) in the Irish et al. (2011a) study, serves to constrain the focus to purely episodic recall in AD. While this approach has proved extremely useful for studying strictly episodic components of ABM narratives, the resultant flat profiles in AD are also revelatory in this context. The disappearance of temporal gradients during ABM retrieval, following the parsing of semantic from episodic details, suggests that semantic knowledge represents a sizeable proportion of remote memory content in AD. This observation meshes well with the view that the episodic and semantic memory systems are invariably interlinked (Greenberg and Verfaellie, 2010), and that episodic memory requires binding of contextual elements within existing frameworks of conceptual knowledge (Reder et al., 2009). The characteristic loss of episodic memory, thus prompts the AD patient to sample intact semantic and gist-based knowledge to guide their retrieval effort, as this approach represents the most accessible and efficient route of access (Greenberg and Verfaellie, 2010; Szpunar, 2010a).

## Autobiographical Memory Retrieval in Semantic Dementia

**Figure 2:**
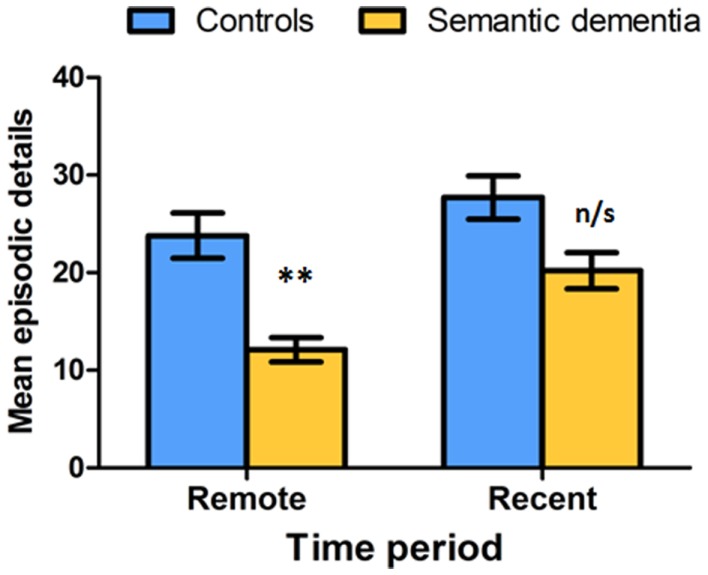
**Differential impairment of remote versus recent autobiographical memory in semantic dementia compared to healthy controls during free recall on the Autobiographical Interview (AI)**. Error bars represent standard error of the mean. **P < 0.0001; n/s, not significant. Data adapted from Irish et al. (2011a).

The investigation of profiles of ABM in SD has elucidated the impact of progressive semantic memory deterioration on episodic memory retrieval. Studies of ABM in SD have yielded inconsistent results, with most pointing to the converse profile to that characteristically seen in AD, namely a reverse temporal gradient, or more accurately, a step function (Hodges and Graham, 2001). The step function describes the observation of relatively preserved recent period retrieval in contrast with impairments in the recollection of memories from more remote epochs (Graham and Hodges, 1997; Nestor et al., 2002; Piolino et al., 2003; Hou et al., 2005; Matuszewski et al., 2009; Irish et al., 2011a; see Figure 2). The precise underpinnings of relatively intact recent period retrieval in SD remain contentious. This effect has been interpreted as reflecting preserved anterograde processes and encoding and retrieval mechanisms (Adlam et al., 2009; Matuszewski et al., 2009). Further, it has been suggested that recent memories encompass more sensory-perceptual elements rather than overgeneral or semanticized information (Hodges and Graham, 2001; Nestor et al., 2002), with SD patients relying on such perceptual features for recent retrieval. The step function profiles of ABM in SD offer further insights into the possible semanticization of episodic memories with repeated rehearsal and the passing of time. The repeated recollection and rehearsal of remote memories allows for the abstraction of the gist of the episode without its accompanying sensory-perceptual details (Rosenbaum et al., 2001), resulting in a largely schematic account of the formerly evocative event. By this view, remote memory deficits in SD reflect a loss of semantic information that is integral to the memory trace (Westmacott et al., 2001). Interestingly, McKinnon et al. (2008) reported an elevation of external (non-episodic) details in concert with a reduction in internal (episodic) details during ABM retrieval in SD. This effect was interpreted as reflecting a relative sparing of generic autobiographical information as well as the provision of tangential details. Thus, while the deterioration of semantic knowledge impinges on the capacity for successful ABM retrieval in SD, semantic elements relevant to the retrieved event are often present within the patients’ ABM narratives. Notably, the ability to retrieve personal semantic and overgeneral autobiographical information from their past appears relatively preserved in SD (Greenberg and Verfaellie, 2010).

It should be noted, however, that a number of studies have failed to replicate the step function during ABM retrieval in SD (Westmacott et al., 2001; Moss et al., 2003; McKinnon et al., 2006; Maguire et al., 2010a). Such inconsistencies in the literature may stem from the methods used to probe ABM retrieval. It is notable that these studies all used non-verbal stimuli, such as family photographs, to elicit ABMs. Importantly, the use of non-verbal stimuli in SD tends to produce a flat profile, with recent and remote memories recalled equally well (Westmacott et al., 2001; Moss et al., 2003; Maguire et al., 2010a; Greenberg et al., 2011). This finding resonates with the proposal that SD patients may harness perceptual features of such visual cues to bypass their profound verbal and language impairments, enabling them to access sensory-perceptual details at a higher level in the ABM system (Conway, 2001; Nestor et al., 2002). The accessibility of perceptual details represents a plausible mechanism underlying preserved recent memory, given that SD patients demonstrate an intact capacity to retrieve sensory-perceptual details during recent, but not remote, ABM retrieval (Irish et al., 2011a), and have been shown to perform normally on sensory-perceptual processing tasks, at least, when feature ambiguity is low (Barense et al., 2010). Critically, the salience of ABMs in SD following the provision of such non-verbal cues speaks to the nature of the interdependence between semantic memory and ABM. When SD patients attempt to retrieve a remote event during traditional verbally loaded ABM tasks, their ability to access relevant perceptual details is hampered by their severe semantic impairment, with the ABM search terminating at the level of non-specific episodes or repeated events in the ABM system (Conway, 2001). Evidence from the domain of ABM therefore underscores the proposition that semantic memories form the basic foundation necessary for retrieval of complex and detailed episodic memories (Greenberg and Verfaellie, 2010). Of note, the Serial-Parallel-Independent (SPI) model proposed by Tulving (1995) has long held that information first enters episodic memory via semantic memory. This model resonates with our view emphasizing the interdependence between these memory systems, and accords with studies demonstrating that impairment in the semantic framework adversely affects the acquisition of new episodic memories in the verbal modality (Ween et al., 1996; Graham et al., 2000). In concert with the progressive deterioration of semantic memory in SD, we see the gradual erosion of episodic ABMs (Maguire et al., 2010a). Collectively, these findings reinforce the view that ABMs necessarily contain, and may critically rely upon, abstracted, supramodal representations of perceptual experiences, which in turn support the sophisticated act of self-projection backwards in time to remember the past (Binder and Desai, 2011; Irish et al., 2012c).

The evidence reviewed here indicates that semantic concepts form an integral component of episodic autobiographical memories, and accords with the conceptualization of autobiographical and semantic memory as opposite ends of a contextual continuum (Kihlstrom, 1984; Burianova et al., 2010). Despite well documented differences in specificity, emotional valence, and contextual detail between these memory types, it is apparent that ABM retrieval relies heavily upon the integrity of semantic information, with the converse observation that semantic memory relies on contextual and episodic components also holding true (Gilboa, 2004; Burianova et al., 2010). Thus, evidence from the study of neurodegenerative conditions serves to reinforce the view that the retrieval of autobiographical memories invariably involves a “synergy between semantic memory and contextual information” (Greenberg and Verfaellie, 2010, p. 749).

## Imagining the Future – Future Oriented Thought

The arena of future oriented thought has undergone a dramatic surge of research activity within the last few years, with growing evidence in favor of a link between remembering the past, imagining the future, and engaging in mental simulation processes (Addis et al., 2007; Hassabis and Maguire, 2007; Hassabis et al., 2007; Schacter et al., 2012). The capacity to imagine specific events in the future has been shown to rely on a number of important component processes including the retrieval of sensory-perceptual episodic details, specificity, fluency, and phenomenological elements such as introspective processes, and the apprehension of subjective time (D’Argembeau et al., 2010). Two prominent theories have been proposed regarding the process by which humans engage in imagining future events. The scene construction hypothesis contends that the capacity to mentally generate and maintain a complex scene within a coherent spatial context represents a critical process which underpins a wide range of constructive processes, such as remembering the past, imagining the future, as well as atemporal and hypothetical simulation (Hassabis and Maguire, 2007, 2009). In contrast, the constructive episodic simulation hypothesis (Schacter and Addis, 2007a,b) holds that the extraction of episodic details from past memories, and their flexible recombination, is fundamental to the successful generation of novel future scenarios. Notably, the discovery that the capacity to envisage future events relies on the same neural machinery as retrieval of autobiographical events from the past (Addis et al., 2007; Szpunar et al., 2007; reviewed by Verfaellie et al., 2012) has proved influential in the resulting conceptualization of future oriented thought. Demonstrations of comparable activity across past and future conditions in the medial temporal lobes, anteromedial prefrontal cortices, posterior cingulate and retrosplenial cortex, lateral parietal and temporal areas has led to the hypothesis that a common “core” network underlies these capacities (Schacter et al., 2007, 2012; see Figure 1). In turn, the largely overlapping neurobiological substrates of past and future modes of thinking have led to the proposition that the capacity to mentally project oneself forward in subjective time is intimately linked to the ability to remember the past (Addis et al., 2007). Unsurprisingly, a sizeable proportion of studies have focused on the episodic component of future oriented thought, with the effects evident even down to the nomenclature of this construct (“episodic future thinking,” Atance and O’Neill, 2001; Klein, 2013) although an episodic-semantic neutral conceptualization has recently been proposed (Stocker, 2012). Under the constructive episodic simulation hypothesis, a fundamental feature of the episodic memory system is its inherent constructive flexibility, which permits the creation of novel events not previously experienced (Schacter and Addis, 2007a,b). Damage to the episodic memory system, therefore, is expected to preclude the ability to mentally simulate future events.

## Parallel Deficits Across Past and Future Contexts in Alzheimer’s Disease

The detection of equivalent deficits across past and future conditions in a range of clinical conditions, including AD (Addis et al., 2009), Mild Cognitive Impairment (Gamboz et al., 2010), schizophrenia (D’Argembeau et al., 2008), and depression (Williams et al., 1996) has strengthened the putative relationship between autobiographical retrieval of the past and simulation of future events. In the study by Addis et al. (2009), patients with AD were found to exhibit marked difficulties in envisaging future events. Importantly these future thinking deficits correlated strongly with retrieval of past events. A recent study investigated the neural correlates of future thinking dysfunction in AD using voxel-based morphometry of structural MRI scans, and corroborated the close correspondence between episodic memory deficits and a compromised capacity for future thinking (Irish et al., 2012a). Crucially, parallel deficits across past and future contexts in AD were associated with disruption to key nodes of the core ABM network, notably the posterior cingulate cortex and the frontal poles. It seems likely that episodic memory dysfunction largely underpins the gross deficits exhibited by AD patients when they are attempting to envision their personal future. Accordingly, AD patients may rely on accessible abstracted semantic representations during future simulation, resulting in gist-based and overgeneral constructions. One area that remains underexplored to date concerns the potential role of scene construction processes as a contributory factor in future thinking dysfunction in AD. Irish et al. (2012a) documented that atrophy in the posterior cingulate cortex, and posterior parahippocampal gyrus correlated significantly with future thinking dysfunction in AD, regions which have been strongly implicated in the construction of spatially integrated scenes (Hassabis et al., 2007). Given the neural regions implicated in future thinking deficits in AD, it is therefore reasonable to assume that scene construction abilities will also be compromised in these patients. Thus, it remains unclear whether the prominent future thinking deficits observed in AD correspond to an impaired capacity for past retrieval, or difficulties with the construction of spatially coherent scenes, or perhaps, more likely, a confluence of multiple deficits arising from widespread neuronal damage to the core network.

## The Arrival of Semantic Memory to the Future Thinking Literature

Greenberg and Verfaellie’s (2010, p. 750) observation that “semantic memories are the basic material from which complex and detailed episodic memories are constructed” seems remarkably fitting when considered in relation to future oriented thinking. Research within the field of future thinking, however, has tended to eschew the possible contribution of semantic memory in favor of focusing on how future thinking may relate to the integrity of the episodic memory system (Klein, 2013). An early study of mental time travel in patient D.B. (Klein et al., 2002) served to reinforce the classic distinction between dissociable systems mediating episodic and semantic past and future thinking. Patient D.B. displayed profound deficits in his recollection of personal events from his past (episodic memory), which, in turn, impinged on his ability to project himself into the future. By contrast, D.B.’s semantic memory was largely preserved, enabling him to remember and to simulate events within the public, non-personal (semantic) domain (Klein et al., 2002). These findings served to reinforce previous views on the dissociation between episodic and semantic memory systems, and pointed toward distinct temporal divisions between what Klein (2013) termed “lived time” for the re-experiencing of the personal past, versus a “known time” in which semantic knowledge is drawn upon to enable temporal projection to the impersonal past.

Up until recently, the traditional heuristic of treating episodic and semantic memory as dissociable systems persisted into the future thinking domain. Klein (2013, p. 66) notes that as the relationship between episodic memory and future orientated thought has strengthened, researchers “simply may have overlooked the possibility that different types of memory contribute to future oriented temporal experience.” A number of anomalies emerged within the literature, whereby experimental findings could not be adequately subsumed under models that exclusively emphasized the role of episodic memory in future oriented thought. In an fMRI study of past and future oriented thinking in personal (episodic) and non-personal (semantic) domains, Abraham et al. (2008b) reported functional dissociations between past and future, and between personal versus non-personal conditions. Importantly, significant engagement of semantic regions, including the inferior temporal gyrus and temporal poles, was observed irrespective of temporality or self-referential condition. Most notable is the observation that patients with developmental amnesia, as a consequence of hippocampal damage, displayed in some instances a preserved capacity to construct future experiences (Maguire et al., 2010b; Hurley et al., 2011; Mullally et al., 2012). Explanations for this relative preservation of future thinking abilities included the possibility that such patients could harness residual hippocampal function to support future projection (Maguire et al., 2010b; Mullally et al., 2012), but also the suggestion that semantic knowledge is an important facet of such imagined scenarios (Cooper et al., 2011; Hurley et al., 2011).

## A Compromised Capacity for Future Thinking in Semantic Dementia

While previous studies pointed to the possible contribution of semantic memory for future oriented thought, the first empirical demonstration that both episodic and semantic memory systems need to be functional to facilitate future thinking has emerged only recently. Duval et al. (2012) investigated the ability of patients with SD to form self-representations across past, present, and future contexts. While patients with SD demonstrated an intact capacity to retrieve recent self-representations, a striking impairment was observed when these patients envisaged their possible future selves. This deficit manifested in a marked inability to construct future self-images or to provide relevant contextual details to support their conception of their future selves (Duval et al., 2012). The authors concluded that personal semantic information therefore makes an important contribution to future oriented self-projection.

**Figure 3:**
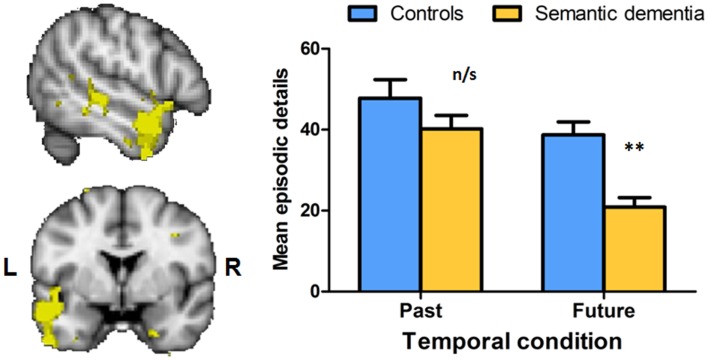
**Deficits in future thinking in semantic dementia are exclusively associated with gray matter intensity decrease in the left anterior temporal lobe, as revealed by voxel-based morphometry analyses**. Clusters are significant at *P* < 0.001 and overlaid on the Montreal Neurological Institute standard brain. Error bars represent standard error of the mean. **P < 0.0001; n/s, not significant. Figure adapted from Irish et al. (2012a).

The asymmetric impairment of future with respect to past oriented thought was corroborated by another study that explicated the neural underpinnings of episodic and semantic future thinking impairments in SD (Irish et al., 2012a). Participants were required to remember three spatiotemporally specific events from the preceding year, and were asked to envisage three novel future events that might occur within the next year. Using the AI scoring procedure, a marked incapacity for future simulation was found in SD, despite their relatively intact retrieval of recent episodic events. Voxel-based morphometry analyses revealed that these striking future thinking deficits in SD robustly correlated with brain regions known to underpin semantic representations (Visser et al., 2010), namely the left anterior inferior temporal gyrus and the bilateral temporal poles (Irish et al., 2012a; see Figure 3). Crucially, these results suggest that the retrieval of past episodes alone is not sufficient for the successful simulation of future events (see also Hassabis and Maguire, 2007; Andelman et al., 2010) and that regions beyond the classic episodic memory system are implicated in complex cognitive endeavors of this kind (Binder et al., 2009; Irish et al., 2012c).

## Differential Disruption of Contextual Details during Future Thinking in SD

The specific vulnerability of future simulation in SD is noteworthy as it speaks to the complexity of this cognitive construct. Further, the findings from the SD literature challenge the prevailing view of future simulation, which to date has focused primarily on the role of the medial temporal lobe and episodic memory (Schacter and Addis, 2007a,b; Race et al., 2011). Given the relative preservation of recent episodic memory in SD, and the profound deficits observed when these patients attempt to envisage their futures, it has been suggested that certain types of details may be differentially disrupted as the patient attempts to move from past to future contexts (Irish et al., 2012a). While much of the research impetus to date has focused on the relationship between episodic memory and future simulation, the extent to which the content of imagined future scenarios reflects elements of episodic memories remains unclear (Szpunar, 2010a). A recent analysis of the types of contextual details reported during future simulation in SD has offered important insights into the component processes underlying the capacity for future oriented thought. Irish et al. (2012b) dissected the types of contextual details embedded within past and future narratives using the AI scoring protocol, and investigated the profiles of contextual details for internal (episodic) and external (non-episodic) subscales. The fine-grained analysis of contextual details in SD has previously pointed to the disruption of recent spatiotemporal and emotional internal details during autobiographical retrieval (Irish et al., 2011a), whereas external details have been shown to be uniquely elevated in this group (McKinnon et al., 2008). Importantly, Irish et al. (2012b) reported that SD patients’ generation of internal event details (i.e., those details conveying the crux of the episode) showed an asymmetric profile (past > future effect), whereby recent past retrieval was within Control levels yet such details were profoundly impaired exclusively in the future condition. In contrast, SD patients demonstrated a future > past effect for external event details, that is, those details which are believed to reflect non-episodic, or semantic, information (Irish et al., 2012b).

The asymmetric decline of internal, and concurrent elevation of external, details during future simulation in SD mirrors that previously demonstrated in healthy aging for retrieval of past autobiographical memories (Levine et al., 2002), and simulation of future events (Gaesser et al., 2011) albeit to an exaggerated degree. Of significance here, however, is the observation that while internal event details can be readily extracted from the past in SD, and remain available during the process of simulation, the construction of a future event ultimately fails. This finding underscores previous observations that thinking about the future necessarily draws upon contributions from both episodic and semantic memory (D’Argembeau and Mathy, 2011; Klein, 2013). In parallel with the failure to utilize readily available episodic details from the past, is the observation that SD patients produce a preponderance of off-target information not directly relevant to the event being simulated. At first glance, the over-production of non-episodic content in a cohort typified by marked semantic deficits seems counterintuitive, however, Irish et al. (2012b) note that such external details, in fact, represent off-target retrieval from unrelated past episodes. Thus the information provided by the SD patients is largely episodic, albeit unrelated to the event being simulated.

These recent findings are important as they resonate with a review article which questioned whether the strict reliance on the contents of episodic memory represents the most efficient route to construct future scenarios (Szpunar, 2010a). By this view, Szpunar (2010a) reasons that the contribution of episodic and semantic elements during future simulation will invariably depend upon the accessibility of information that is relevant to the event of interest. Crucially, the content of any future simulation will reflect the information that is most readily accessible (Kahneman and Tversky, 1982; Szpunar, 2010a,b). Healthy individuals are likely to draw upon abstracted representations and such representations should, in general, be more accessible than specific one-off episodic representations (Szpunar, 2010a). In contrast, certain scenarios may exist in which episodic representations of specific events may represent the most efficient mode for future thinking. If we consider the findings from the SD patients in the Irish et al. (2012a,b) series, it becomes evident that the information most accessible to these patients is that of unique recent episodic occurrences and more general repeated events or “repisodes” in the absence of general conceptual semantic knowledge.

## The Semantic Scaffolding Hypothesis

If episodic details represent the most accessible and efficient means for SD patients to construct a future scenario, why then are these details so vulnerable during future simulation? In line with current views emphasizing the importance of episodic and semantic contributions during future thinking, the findings from SD studies offer compelling evidence that the disintegration of the conceptual knowledge base adversely affects the ability to construct events in the future (Irish et al., 2012a). In this light, the recently proposed semantic scaffolding hypothesis is particularly pertinent, whereby semantic knowledge appears to provide a framework or scaffolding which facilitates past retrieval and future thinking (Greenberg and Verfaellie, 2010; Irish et al., 2012a). This semantic scaffolding hypothesis is relevant to the finding that a loss of semantic knowledge precludes the ability to simulate novel future events. In SD, the successful extraction of sensory-perceptual details from recent events occurs in the absence of the necessary conceptual framework to impart overall structure to the scenario (Irish et al., 2012b). If extraction of episodic details from the past represents the building blocks of future simulations, any attempt to construct a coherent scenario without the necessary scaffold or framework, results in the provision of a series of unrelated mini-events. Essentially, the episodic details cannot be integrated within the appropriate schema or abstracted representation from semantic memory. One further issue to disentangle, in this regard, is at which point in the simulation process semantic memory becomes pivotal? SD patients may not possess the relevant abstracted semantic representations to facilitate the construction of future events; however, a second possibility is that the loss of conceptual knowledge in SD also adversely impacts on the integrative mechanism necessary to bind episodic details into a coherent simulation. The final product of a successful future simulation likely comprises elements of various episodic and semantic details that are flexibly recombined to create an integrated and coherent representation of a specific future event (Szpunar, 2010a; Addis et al., 2011). It remains unknown, however, whether the amodal loss of semantic knowledge in SD precludes the recombination of episodic details from past retrieval into a coherent novel scenario, although this proposal represents an intriguing and plausible explanation for the elevation of unrelated event details in this group (Irish et al., 2012b). Further work is necessary to elucidate the precise mechanisms of detail recombination and, specifically, whether the reconfiguring of past details entails some form of semantic associative processing. It is possible that recombination may reflect a two-step process, in which firstly semantic associations between disparate details are made, drawing on abstracted representations that are accessible contingent on the specific task requirements, following which a process of integrative binding is required to flexibly recombine these details together into a coherent spatiotemporal framework. Determining the precise constituent elements of recombination within semantic frameworks represents an important line of enquiry for future research.

## Semantic Knowledge and Novelty of Future Simulations

Semantic memory may be particularly important for the simulation of novel future events, in which no prior experience can be drawn upon from episodic memory. By its nature, semantic memory can be generalized to many different contexts (Mion et al., 2010), thus providing undifferentiated conceptual information that can be drawn upon to facilitate novel event construction (Binder and Desai, 2011; Irish et al., 2012a). The novelty of the simulated event becomes of paramount importance when we consider the profound difficulties experienced by patients with SD in envisioning events that have not occurred previously. Irish et al. (2012a) found that 80% of future events described by SD patients represented events that had been previously experienced in their entirety. Simply put, the SD patients demonstrated a marked propensity to sample past episodes and to recapitulate these events into the future condition, despite explicit task instructions requiring them to generate novel events not previously experienced. The severe semantic impairment in SD, therefore, disrupts the capacity for novel event generation, manifesting in scenarios that have been recast from intact past memories (Irish et al., 2012b). Recasting of the past is an intriguing phenomenon in SD, and suggests that the construction of novel events critically relies on the harnessing and deployment of semantic knowledge. By this argument, events which occur within familiar and repeated spatiotemporal contexts may be more readily accessible in SD, as the patients can successfully retrieve prior instances within such familiar contexts (Graham et al., 1999; Irish et al., 2011a). It may be that the envisaging of novel scenarios, for which one has little to no prior episodic experience, heavily taxes the semantic memory system, requiring the individual to draw on general world knowledge to guide them in their constructive endeavor. In SD, however, the progressive deterioration of the semantic memory system renders such conceptual knowledge inaccessible, causing the patient to overly rely on previously encountered scenarios, and ultimately resulting in the recapitulation of past events into the future condition.

It has recently been suggested that the creation of a suitable “situation model” represents a crucial process to facilitate the retrieval of past, and simulation of future, events (Ranganath and Ritchey, 2012). The successful formation of a situation model is, in turn, posited to depend on the integrity of a posterior medial cortical system centered on the posterior parahippocampal cortex and retrosplenial cortex (Ranganath and Ritchey, 2012). This view corroborates previous fMRI studies of healthy individuals engaging in future simulation, in which the importance of posterior regions including the posterior cingulate cortex and parahippocampal cortex in the activation of well-known contextual settings has been emphasized (Szpunar et al., 2009). Notably, posterior brain regions remain relatively preserved until later in SD. These posterior regions offer a potential neuroanatomical signature for the harnessing of familiar contexts effect that is reliably demonstrated in future thinking studies in SD. Accordingly, SD patients draw upon the accessible situation model but cannot integrate or update this model to create a novel scenario, and inevitably recapitulate overgeneral or previously experienced events during future simulation. Further exploration of this recasting of familiar contexts effect in SD is clearly warranted, in particular how this phenomenon is related to the observation of an intact capacity to generate “repisodes” (Neisser, 1981) during ABM retrieval (Greenberg and Verfaellie, 2010). Likewise, an obvious outstanding area of research relates to whether the amodal loss of semantic knowledge in SD precludes the construction of spatially coherent scenes, in line with the scene construction theory advanced by Hassabis and Maguire (2007) and Hassabis et al. (2007). Unraveling the contribution of semantic memory to scene construction processes will be essential in clarifying the precise role of conceptual knowledge in memory and imagination.

## Concluding Remarks

We conclude this review at an exciting juncture in episodic memory research. What has become evident is the incontrovertible extent to which episodic and semantic memory interact during complex forms of past and future oriented thinking, and the limitations of couching such endeavors within the traditional taxonomy of episodic and semantic memory as dissociable systems. The studying of complex cognitive processes in neurodegenerative conditions has proved critical for explicating how episodic and semantic elements may work in concert during autobiographical retrieval and future simulation, yet much remains to be elucidated. We suggest that concerted efforts are warranted to disentangle and elucidate the precise contributions of each memory system to constructive recollective and simulative endeavors, which in turn will illuminate our understanding of the episodic memory system of the brain.

## Conflict of Interest Statement

The authors declare that the research was conducted in the absence of any commercial or financial relationships that could be construed as a potential conflict of interest.
